# Changes in Echocardiographic Parameters among Beninese Soccer Referees during the Division 1 Championship in 2016

**DOI:** 10.1155/2018/6024574

**Published:** 2018-11-07

**Authors:** Quenum Coffi, Sonou Arnaud, Gouthon Polycarpe, Ahissou Hyacinthe, Messan Folly, Nouatin Basile, Hounkponou Murielle, Houénassi D. Martin

**Affiliations:** ^1^Laboratory Sports Performance, Health and Evaluation, National Institute of Youth, Physical Education and Sport (INJEPS), University of Abomey-Calavi (UAC), 01 P.O. Box 169, Porto-Novo, Benin; ^2^Department of Cardiology, Departmental Teaching Hospital of Ouémé-Plateau, University of Abomey-Calavi (UAC), 01 P.O. Box 169, Cotonou, Benin; ^3^Laboratory of Enzymology and Biochemistry of Proteins, College of Science and Technology, University of Abomey-Calavi (UAC), 01 P.O. Box 188, Cotonou, Benin; ^4^Laboratory Respiratory Physiology and Functional Exploration, National Institute of Youth, Physical Education and Sport (INJEPS), University of Abomey-Calavi (UAC), 01 P.O. Box 169, Porto-Novo, Benin; ^5^Department of Cardiology, National Teaching Hospital of Cotonou, University of Abomey-Calavi (UAC), 01 P.O. Box 827, Cotonou, Benin

## Abstract

**Introduction:**

The goal of this study was to describe the echocardiographic parameters of soccer referees and to examine the changes in these parameters after a period of intensive physical exercise.

**Methods and Patients:**

We conducted a prospective study that included Beninese soccer referees. The study of the geometry and function of the left ventricle (LV) was made at the beginning and end of the national Division 1 championship, which was held during the course of 10 weeks.

**Results:**

There were 37 referees included in this study; 20 at the national level (G1: 27.8 ± 6.6 years) and 17 at the international level (G2: 32.1 ± 6.4 years). Dimensions of the LV were normal for all the referees. At the beginning of the championship, 51.3% of the referees had a normal LV geometry, 37.8% had concentric remodelling, 2.7% had concentric hypertrophy, and 8.1% had eccentric hypertrophy. In the group of referees with normal LV geometry, a modification in concentric remodelling at the end of the championship was seen in 30% of the referees in G1, 33.3% of the referees in G2, and 31.6% of the whole sample. In the group of subjects who presented concentric LV remodelling, a modification in the normal geometry was observed in 37.5% of those in G1, in 0% of those in G2, and in 21.4% of the whole sample. The cases of LV hypertrophy showed no change regardless of the group considered. An LV ejection fraction of more than 50% and an E/E′ ratio less than 8 were found in all referees.

**Conclusion:**

All the referees studied had normal cardiac morphology and function. The intensity of the physical load was insufficient to impact this morphology.

## 1. Introduction

Football refereeing is a highly demanding activity that requires great physical qualities. During a football match, the central referee runs an average distance of 10 to 13 kilometres and the assistant referee runs 6 to 8 kilometres, with 150 accelerations and 800 to 1000 changes of direction [1].

Changes in the heart structure and function following periods of sport training are grouped under the term* athlete*'*s heart*. This condition is mainly bradycardia and in echocardiography, a proportionally more marked dilatation of the left ventricle (LV) cavity than the wall hypertrophy. Indeed, markedly dilated LV chambers (>60 mm) were most common in athletes with higher body mass and those participating in endurance sports (cycling, cross-country skiing, and canoeing) [2]. As an example, a programme of 18 weeks of intensive training caused an increase in LV end-diastolic (P = 0.0001) and end-systolic volumes (P < 0.0001) among 91 athletes at the national and international level [3]. In addition to the modifications previously reported, cases of sudden death have been reported in high-level training athletes belonging to the elite in their countries [4, 5]. Although intensive sport practised by a healthy athlete does not increase his cardiovascular risk, high-level practice can provoke sudden cardiac death in cases of unknown prior cardiac disease. Therefore, cardiovascular events observed in athletes are often due to unknown cardiac disease revealed by chronic intensive exercise. The impact of sport practice on the heart according to the level and type of exercise and population of athletes has been the subject of many publications aimed at distinguishing physiological from pathological cardiovascular modifications [6, 7].

To date, cardiovascular changes associated with the practice of football refereeing in West African countries have been poorly documented. This study was therefore initiated to describe the echocardiographic parameters of a population of top-level Beninese soccer referees and to identify the modifications in these parameters at the end of a period of exposure to a high-level sport activity, i.e., a competition.

## 2. Materials and Methods

We conducted a prospective study in the Republic of Benin (towns of Porto-Novo and Cotonou) during the Division 1 (D1) soccer championship of the 2016 season. The population studied included referees registered on the official list of the Referee's Central Commission in Benin. Active male referees (FIFA, Confederal, or federal confirmed, and trainees with at least one year of seniority) belonging to the regional commissions of the south and southeast were included. The sample was divided into 2 groups: G1 was at the national level (confirmed federal referees and trainee referees), and G2 was at the international level (FIFA and Confederal referees). Subjects who were sick during the study or during the two weeks preceding the championship were excluded. The sample was constituted using the nonprobabilistic method and the exhaustive technique.

**Figure 1:**
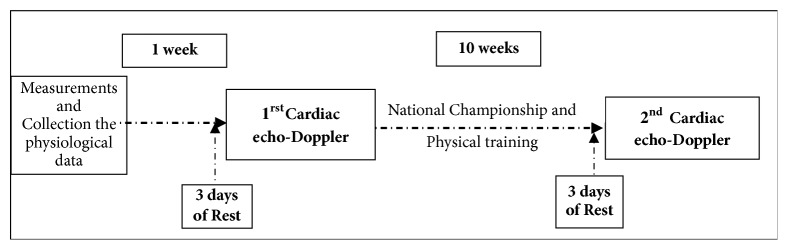
Study design and diagram of data acquisition among soccer referees of national and international level in Republic of Benin.

The study variables were the following:

(i) General characteristics: weekly time of training.

(ii) Anthropometric parameters: weight, height body surface, and body mass index (BMI), obtained by calculating the weight in kg divided by the height in square metres.

(iii) The LV geometry [8]: end-diastolic diameter (LVEDD), indexed end-diastolic diameter (LVEDDi), indexed LV mass (LVMi), interventricular septal thickness during diastole (IVSd), and LV posterior wall thickness during diastole (LVPWd). We calculated the relative wall thickness (RWT = double the LVPWd divided by the LVEDD) to define the normal LV geometry (RWT ≤ 0.42 and LVMi ≤ 115 g/m^2^), LV concentric remodelling (RWT > 0.42 and LVMi ≤ 115 g/m^2^), LV concentric hypertrophy (RWT> 0.42 and LVMi> 115 g/m^2^), and LV eccentric hypertrophy (RWT ≤ 0.42 and LVMi> 115 g/m^2^).

(iv) The LV function: cardiac flow ($\dot{Q}c$), stroke volume (SV), ejection fraction (EF), velocity of the E and E' waves at transmitral filling, and the E/E' ratio. Since FIFA physical tests and resting ECG are routinely conducted once a year at the beginning of the season and then resumed before each grouped international competition, a physical examination and resting electrocardiogram were not performed in our sample of referees specifically for this study. In our study, only anthropometric measurements and those concerning LV morphology and function were taken twice: 7 days before the beginning and 7 days after the end of the D1 championship, which lasted 10 weeks (Figure 1). The referees' physical training session usually consisted of three parts, covering a duration of 120 minutes. The first part was the warm-up and included running three or four laps in small strides around the athletic track, followed by some specific stretching exercises for the lower limbs. In the second part, the referees completed 10 laps of the track intermittently (150 m of running in 30 s and 50 m of walking in 35 s, repeated 20 times). Finally, the session ended with 3 to 5 minutes of strengthening exercises for the arm and abdominal muscles, followed by a recovery time based on stretching.

The weekly training time was indicated by the referees and their individual number of supervised matches, according to the Referee's Central Commission. An echocardiograph SSI-8000 (SONOSCAPE, China), handled by a trained cardiologist, was used to collect the echocardiographic data. After 3 successive measurements, the average values were retained. All the referees gave their informed and written consent to participate in the study.

Prior agreement from the Scientific Committee of Sciences and Techniques of Physical and Sports Activities (University of Abomey-Calavi) and the South Regional Commission of the Referees was obtained.

The recorded data were treated with the Statistica software (Stat Soft Inc., Version 10.0). Descriptive statistics, such as number (n), mean value (m) ± standard deviation (s), or standard error of the mean (SEM), were calculated for each studied variable. Student's paired t-test was used for intragroup comparisons (first* versus* second time measurements), and Student's unpaired t-test was used for intergroup comparisons (national* versus* international level referees). The level of significance of the statistical tests was set at p < 0.05.

## 3. Results

### 3.1. General Characteristics and Anthropometric Parameters

The sample comprised 37 referees, including 20 in the G1 group and 17 in the G2 group. The referees, all levels combined, officiated/directed 5 ± 2 matches during the championship. They were on average 29.8 ± 6.8 years old. In the G1 group, the referees' weekly training was 4.2 ± 0.2 hours* versus *8 ± 0 hours for the G2 group (P < 0.0001) before the championship, and there was no change during the competition period. The anthropometric parameters of the G1 and G2 groups at the beginning of the championship are presented in Table 1. At the end of the championship, the BMI decreased significantly in the whole sample (22.7 ± 0.4 kg.m^−2^* versus *22.4 ± 0.3 kg.m^−2^; P = 0.003).

### 3.2. Changes in Cardiac Morphology

All the examined referees had an LVEDD lower than 60 mm and a parietal thickness (IVSd or LVPWd) lower than 14 mm.  Table 2 shows the mean values of the LV dimensions in the G1 and G2 groups before the beginning and at the end of the championship. The LVEDDi was an average of 26.8 ± 0.1 mm/m^2^ G1 and 27.3 ± 0.1 mm/m^2^ in G2. The LVEDDi dropped significantly in G2 at the end of the championship (P = 0.002). Concerning the types of LV geometry, 51.3% of the subjects had normal geometry, 37.8% had concentric remodelling, 2.7% had concentric hypertrophy, and 8.1% had eccentric hypertrophy before the championship. At the end of the competition period, the referees who had ventricular hypertrophy did not show any changes in their geometry.  Table 3 presents the values related to the cardiac morphology.

### 3.3. Changes in Cardiac Function

All the examined referees had an LV EF > 50%, and none had an E/E' ratio > 15. In the G1 group (Table 4), decreases were observed in HR_r_ of 2.9% (P = 0.04) and $\dot{Q}c$ of 1.3% (P = 0.02) at the end of the championship. A decrease in the LV EF of 5.3% was noted in G1 at the end of the championship (P < 0.01).

## 4. Discussion

The main objectives of this study were to describe the echocardiographic aspects of Beninese soccer referees and analyse the modifications induced by 10 weeks of national D1 championship game activity.

### 4.1. Methodological Aspects

The difference in age observed between the G1 and G2 referees can be explained by the number of years of experience required to apply at the international level. However, the average age of the G2 referees was lower than that of their top-level European fellow members, which is approximately 40 years [9]. The average age of our entire study sample is comparable to that of national level Spanish referees [10].

This study has two main limitations. The first relates to the lack of an untrained group of healthy males, which would make it possible to specify the potential effect of the training in the studied referees, and the second limitation concerns the small sample size. We attempted to recruit age-matched adults, but only three people agreed to participate, so we stopped the recruitment. It was also not possible to increase the study sample size, as we enrolled all available referees.

### 4.2. Anthropometric Parameters

A significant reduction in BMI was noted at the end of the championship in the entire sample. However, this tendency was not recorded in the G2 group, where the amount of weekly training was higher than that in the G1 group. One must keep in mind that these 10 weeks of the championship represents one-third of the duration of the D1 championship in high-performance countries, where soccer teams play this competition during the course of 30 to 32 weeks.

### 4.3. Parameters of Cardiac Morphology

Concerning the imagery of the athlete's heart, reference and normal values of echocardiographic parameters were defined according to the guidelines of the European Society of Cardiology [8]. Taking these reference values into account, we can assume that no pathological value of the left ventricle's morphology was recorded in the two groups of referees. It is known that a regular practice of intensive sport exercise induces echocardiographic changes according to the effort's intensity [3, 11]. In a study by Galanti, the average LVEDD observed among D1 referees was higher than that of the entire group of referees [12]. Among Beninese referees, there was no difference in LVEDDi between the G1 and G2 groups, although the weekly amount of training was higher in the G2 group. Therefore, the higher training level of the G2 referees should be considered insufficient to impact or improve the LVEDDi and therefore the LV geometry. The LV geometry before the championship was dominated by the normal form (51.3%) and by concentric remodelling (37.8%), with only 2.7% of referees having LV eccentric hypertrophy. At the end of the championship, there were no new cases of left ventricular hypertrophy. These observations do not agree with Morganroth's hypothesis that LV eccentric hypertrophy is prevalent among soccer referees [13]. The relatively short duration of the championship in our study (10 weeks) may partly explain this result.

### 4.4. Parameters of Cardiac Function

The heart function of the referees in this study was normal since none of them had an LV ejection fraction lower than 50% [8]. Significant decreases in $\dot{Q}c$ and EF were recorded in the G1 group at the end of the championship, without overreaching the physiological limits.

It is known that 6 weeks of intensive sport training results in an increase in stroke volume due to increased venous return [14]. This parameter did not show a significant change among referees in the current study. We therefore can conclude that the 10 weeks of championship was not intensive enough. Interexamination variability is also a possible explanation for the lack of change.

Bradycardia, a main characteristic of athletes' hearts, was observed in both groups, with the mean resting heart rate lower in the G2 group (55.6 ± 1.5 bpm) than in the G1 group (56.7 ± 1.5 bpm) at the end of the championship. This difference confirms the relation between the level of training and the reduction of the resting heart rate [9].

## 5. Conclusion

At the end of this study on the echocardiographic parameters of 37 elite soccer referees in the Republic of Benin, no pathological changes in the left ventricular morphology or function were observed. After 10 weeks of Division 1 championship, the left ventricular geometry, dominated by the normal type, also did not show any significant changes. A larger series will highlight specific aspects of soccer referees' hearts in West Africa.

## Figures and Tables

**Table 1:** Anthropometric parameters of a sample of soccer referees at the beginning and at the end of the Division 1 championship (Benin, 2016).

	**National level** **(n** = **20)**		**International level** **(n** = **17)**		**whole sample** **n** = **37**	
	At the beginning of the championship	At the end of the championship	At the beginning of the championship	At the end of the championship	At the beginning of the championship	At the end of the championship
**Height (cm)**	170.1 ± 1.4	-	176.4 ± 1.5††	-	173.0 ± 1.1	-

**Body mass (kg) **	65.0 ± 1.8	63.7 ± 1.7	70.9 ± 1.8†	70.6 ± 1.7	67.7 ± 1.3	66.9 ± 1.3

**BMI (kg/m** ^**2**^ **)**	22.5 ± 0.5	22.1 ± 0.5*∗∗*	22.8 ± 0.6	22.7 ± 0.5	22.7 ± 0.4	22.4 ± 0.3*∗∗*

**WTA (H)**	4.2 ± 0.8	-	8.0 ± 0.0	-	5.9 ± 2.0	-

**SIA (year)**	1.8 ± 0.6	-	2.7 ± 0.4	-	2.2 ± 0.7	-

BM: body mass; BMI = body mass index; WTA: weekly training amount; SIA: seniority in the arbitration; †: significant intergroup difference at P < 0.05; ††: significant intergroup difference at P < 0.01; *∗∗*: significant.

**Table 2:** Morphological parameters of cardiac left ventricle in a sample of soccer referees at the beginning and the end of the Division 1 championship (Benin, 2016).

	**National level** ** (n** = **20)**		**International level** ** (n** = **17)**	
	At the beginning of the championship	At the end of the championship	At the beginning of the championship	At the end of the championship
**IVSd (mm)**	9.9 ± 0.0	10.1 ± 0.0	10.4 ± 0.1	11.0 ± 0.0
**LVEDD (mm)**	45.8 ± 0.4	45.0 ± 0.4	49.1 ± 0.4	47.3 ± 0.4
**LVEDDi (mm/m** ^**2**^ **)**	26.8 ± 0,1	26.6 ± 0.1	27.2 ± 0.1	26.4 ± 0.0*∗∗*
**LVPWd (mm)**	8.9 ± 0.0	8.3 ± 0.0	8.7 ± 0.0	8.9 ± 0.0
**LVM (g)**	149.1 ± 7.6	140.1 ± 6.2	170.8 ± 10.6	170.1 ± 9.6

IVSd: thickness of the interventricular septum in diastole; LVEDDi: indexed left ventricular end-diastolic diameter; LVPWd: thickness of the left ventricular posterior wall in diastole; LVM: left ventricular mass; LVMi: indexed left ventricular mass; *∗∗*: difference between beginning and end of the championship, significant at P < 0.01; ††: intergroups difference, significant at P < 0.01; †††: intergroups difference, significant at P < 0.001; duration of the championship of Division 1 in 2016 = 10 weeks.

**Table 3:** Distribution of the referees according to the types of left ventricular geometry which showed modification at end of the championship (Benin, 2016).

	**At the beginning of the championship**	**At the end of the championship**	
**Whole group **	NG = 19	NG = 13 (68.4%)	CR = 6 (31.6%)
	CR = 14	NG = 3 (21.3%)	CR = 11 (78.6%)
**National level**	NG = 10	NG = 7 (70.0%)	CR = 3 (30.0%)
	CR = 8	NG = 3 (37.5%)	CR = 5 (62.5%)
**International level**	NG = 9	NG = 6 (66.6%)	CR = 3 (33.4%)
	CR = 6	NG = 0 (0.0%)	CR = 6 (100.0%)

NG: normal geometry; CR: concentric remodeling.

**Table 4:** Changes in the parameters of the left cardiac function of the studied soccer referees, between the beginning and the end of the Division 1 championship.

	**National level**		**International level**	
	At the beginning of the championship	At the end of the championship	At the beginning of the championship	At the end of the championship
**H** **R**_**r**_ ** (bpm)**	58.45 ± 2.09	56.70 ± 1.46*∗*	56.17 ± 1.64	55.64 ± 1.49†††
$\dot{V}O_{2}$ **max (ml/min/kg)**	55.30 ± 2.82	55.47 ± 2.80	54.83 ± 2.98	56.72 ± 2.96†††
**SV (mL)**	66.05 ± 3.90	63.86 ± 3.17	68.43 ± 3.56	70.21 ± 3.03
$\dot{Q}c$ ** (L/min)**	4.22 ± 0.22	3.92 ± 0.19*∗*	4.12 ± 0.18	4.20 ± 0.15
**EF (**%**)**	69.85 ± 1.39	66.12 ± 0.99*∗∗*	70.46 ± 1.62	70.29 ± 1.38†
**E (m.** **s** ^−1^ **)**	0.90 ± 0.03	0.88 ± 0.03	0.81 ± 0.01	0.85 ± 0.03
**E' (m.** **s** ^−1^ **)**	0.28 ± 0.03	0.29 ± 0.10	0.27 ± 0.03	0.20 ± 0.00*∗*
**E/E'**	3.96 ± 0.36	4.27 ± 0.24	3.59 ± 0.33	4.34 ± 0.21*∗*

HR_r_: resting heart rate; $\dot{V}O_{2}$max: maximum oxygen consumption estimated indirectly by the results of the YoYoIR2 test (Bangsbo et al., 2008); SV: stroke volume; E: speed of transmitral filling in diastole; E': speed of transmitral filling in systole; EF: ejection fraction; HR: heart rate resting; *∗*: difference between values before and those of the championship, significant at p < 0.05; *∗∗*: difference between values before and those of the championship, significant at p < 0.01; †: significant at p < 0.05; ††: significant at p < 0.01; †††: significant at p < 0.001.

## Data Availability

The data used to support the findings of this study are available from the corresponding author upon request.
